# Insights into student participation in a soil physics course during COVID‐19 emergency online learning

**DOI:** 10.1002/nse2.20036

**Published:** 2021-01-25

**Authors:** Briana Wyatt

**Affiliations:** ^1^ Dep. of Soil and Crop Sciences Texas A&M Univ. 2474 TAMU College Station TX 77843 USA

## Abstract

The recent novel coronavirus pandemic led to global changes in higher education as universities transitioned to online learning to slow the spread of the virus. In the United States, this transition occurred during the spring of 2020, and the compulsory shift to online learning led to frustrations from students and instructors alike. I studied student participation during the online portion of a university‐level soil physics course taught in Spring 2020. Participation was quantified using the number of student posts in weekly discussion boards, the number of student views of asynchronous videos, and the number of video views during each week of online instruction. Relationships between video length and number of student views and between student participation and final exam grades were also examined. My findings show that student views of mini‐lecture videos were low and decreased throughout the online learning period. Conversely, views of example problem videos and the number of posts on graded discussion boards were high and remained high throughout the online learning period, suggesting that students were more engaged with online material that affected their grades. I also found that the level of student engagement in online material was positively correlated with higher final exam scores. The findings presented here may be used to improve the development and delivery of online coursework in natural science disciplines, both during current and future emergencies.

## 1 INTRODUCTION

University instructors and students alike were forced to adopt new teaching and learning strategies as courses worldwide were forced online as a result of the novel coronavirus (COVID‐19) outbreak during the 2019–2020 school year (Crawford et al., 2020). In the United States, this rapid transition occurred primarily in the middle of the spring semester. Due to the short transition period between in‐person and online teaching at most U.S. universities, stronger emphasis was often placed on rapidly training instructors on how to utilize the online learning environment than on developing robust online pedagogy (Crawford et al., 2020). In many cases, a lack of instructor familiarity with online learning environments combined with student disinterest in online coursework led to frustrations from students and instructors alike. These frustrations were further compounded by anxiety regarding personal health (Jungmann & Witthöft, 2020), inequitable access to a reliable internet connection (Beaunoyer, Dupéré, & Guitton, 2020), and economic hardship associated with the pandemic (Mann, Krueger, & Vohs, 2020), among other concerns.

At Oklahoma State University, the decision to move all courses online temporarily was made just before students’ spring break holiday (16–20 March) and instructors were given this 5‐day period to prepare to present the following 2 weeks of course content online. Soon thereafter, the decision was made to continue online coursework for the remainder of the semester. For most students, the transition from in‐person class meetings to online learning posed several challenges, especially as instructors were strongly encouraged to avoid synchronous online class meetings to allow students flexibility in when and where they accessed course materials. This asynchronous online model translated into fewer opportunities for instructor–student interaction, decreased instructor feedback from students, and, based on the findings presented here, a decline in student interest in course content.

According to Bristow et al. (2011), who polled more than 800 students at a midwestern U.S. university, students have, on average, reported relatively neutral feelings toward online classes as compared to in‐person instruction. However, a sizeable minority of the student group considered in that same study reported strong negative feelings toward online learning, demonstrating that there is a wide range of student feelings and responses toward even non‐compulsory online coursework. Further, Lyke and Frank (2012) showed that although student performance did not vary significantly between online and in‐person versions of a course, students in the online version of the course reported being less satisfied with both the course and the instructor. My current findings related to student participation in and satisfaction with compulsory COVID‐19 emergency online teaching mirror the reported student dissatisfaction with online learning that has been reported in these, and other, prior studies.

Here, I present an analysis of student online engagement and participation in an introductory university soil physics course during the latter portion of the Spring 2020 semester, when courses were forced online due to the COVID‐19 pandemic. Course enrollment totaled 26, with 15 undergraduate and 11 graduate students from a wide range of disciplines, including agronomy, environmental science, natural resource ecology, entomology, and engineering. Factors studied include the number of students participating in weekly discussion boards; number of views of on‐demand online mini‐lecture, example problem, and answer key videos both at the beginning and end of each video; the number of video views during each week of the online learning period; the relationship between mini‐lecture video length and number of student views; and the relationship between students’ level of online participation and final exam letter grades.

My findings provide several key insights into student behavior during this emergency online learning period: (a) the greatest levels of student participation were found for course content that directly influenced students’ grades; (b) students’ engagement in viewing asynchronous online mini‐lecture videos was generally low and decreased significantly with time; (c) many students who began videos did not watch videos to completion, (d) the number of student views per mini‐lecture video was not related to video length; and (e) the level of student engagement in online course material was positively correlated with higher final exam scores. Overall, my findings indicate that university students have little interest in compulsory online coursework and suggest that a purely asynchronous online teaching model may lead to low student engagement.

**Core Ideas**

- Student views of asynchronous mini‐lecture videos were low and decreased with time.
- Student participation was greatest when grades were influenced.
- Number of student views per mini‐lecture video not correlated with video length.
- Student online participation is positively correlated with higher final exam scores.

## 2 METHODS

### 2.1 Course design prior to online learning

During the in‐person portion of the semester, the course was designed as a flipped classroom. In this model, students were assigned weekly readings from an open source textbook (one chapter per week) to be completed over the weekend. Before the first class meeting of each week, students were expected to make a participation notecard including responses to three prompts: a “star” prompt, which asked the students what their favorite concept was from each chapter; a “question mark” prompt, which asked what questions the students had; and an “arrow” prompt, which asked how the reading material was relevant to the students, either academically or professionally. These notecard responses were graded as participation points and provided the basis of in‐class discussions. Such post‐reading activities have been shown to provide students with the opportunity to review, summarize, and react to assigned readings and also provide instructors with a sense of student comprehension (Aliyeva, 2020).

In‐class discussions based on these notecards, which have been shown to increase student performance in this particular course (Ochsner & Robinson, 2017), were the focal point of class meetings. During these discussions, students would utilize class time to work with partners to increase their understanding of the reading material, answer each other's questions, and ask me questions during class so that all students would have a clear response. Student attendance and participation during the in‐person portion of the semester was high, and although attendance records were not kept, it is estimated that ∼22 of the 26 enrolled students attended each class meeting.

In addition to in‐class discussions and to increase student understanding, students’ problem sets and exams were self‐graded during class meetings. The practice of self‐grading has been shown to increase student motivation and create a sense of responsibility for learning (Strong, Davis, & Hawks, 2010), and is intended to increase students understanding regarding course concepts. During self‐grading, correct answers were shown step‐by‐step to the students, who were able to follow along using their finished work and take notes using a different colored writing implement than the one that was used to complete the assignment or exam. This process allowed students to see the correct answers, to ask questions regarding those answers, and to improve their understanding of course concepts. It also provided insight into which topics students understood, which concepts they struggled with, and helped me determine which material to focus on more closely.

### 2.2 Course design during online learning

After the transition to emergency online learning, the ability of students to communicate with one another and with me decreased substantially, primarily as a result of the need for online classes to be asynchronous. In an attempt to bridge this communication gap, weekly discussion boards were posted in the online learning environment. There, students were expected to post their responses to the three prompts described above after reading each assigned chapter from the textbook, effectively creating a “digital notecard,” which was graded as participation points. This allowed me to identify gaps in students’ understanding, which I often addressed both by responding the comments in the discussion board and with additional instruction in pre‐recorded mini‐lecture videos. Additionally, to earn full participation points and in an attempt to stimulate communication between peers, students were also required to provide feedback to at least one other person's prompt responses.

In addition to the participation discussion boards described above, students also had access to a variety of short, on‐demand videos related to course textbook content, problem set example problems, and answer keys to problem sets and exams. These videos were created to serve as an alternative to the in‐class instruction that occurred before the online learning period, and although the pre‐recorded videos were one‐sided, they presented a thorough examination of the content of each textbook chapter, additional relevant information that was not presented in the textbook, and valuable information regarding how to complete each chapter's problem set.

### 2.3 Measuring student engagement and participation

Student engagement with online coursework during the online learning period was quantified using several metrics, including student participation in weekly online discussion boards; the total number of student views of on‐demand instructional videos, both at the beginning and end of each video; the total number of mini‐lecture video views during each week throughout the online learning period; and the relationship between mini‐lecture video length and number of views. Data regarding each of these metrics were collected after the semester had ended from the learning management system used throughout the online portion of the course. Types of videos included mini‐lecture videos (*n* = 31), example problem videos (*n* = 11), and videos containing answer keys to problem sets and exams (*n* = 4).

The average number of student discussion board posts throughout the online learning period and total number of discussion board posts for each chapter are discussed, as are the average number of student video views overall and for each video type. One‐way analysis of variance (ANOVA) was used to determine significant differences in (a) the total number of video views at the beginning and at the end of videos, (b) the average number of views of different video types, and (c) final exam letter grades based on the number of participation events per student. Additionally, ANOVA was used to identify significant differences in three measures of student participation—average number of mini‐lecture video views, example problem video views, and number of discussion board posts—during each week of the online learning period. Finally, inferences into student motivation are drawn from trends in online course participation during the Spring 2020 COVID‐19 emergency learning period and suggestions are made regarding best practices for future online course development amid the COVID‐19 pandemic.

## 3 RESULTS AND DISCUSSION

### 3.1 Access of on‐demand videos

**FIGURE 1:**
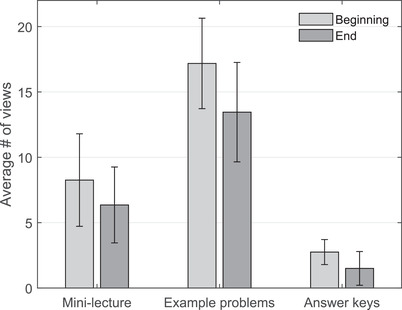
Average number of views of mini‐lecture, example problem, and answer key videos at both the beginning and end of each video. Error bars show 1 SD above and below the mean. When data were paired by video type, significant differences were found between the average number of views at the beginning and end of videos for the mini‐lecture, but not for the example problem or answer key videos. Significant differences were also found between the average number of views at the beginning of each of the three video types

The number of student views of on‐demand videos throughout the online learning period was low, suggesting a lack of interest in compulsory online learning. On average across all on‐demand video types, only 9.9 students (38.1%, standard deviation [SD] = 5.5) began watching these videos and only 7.6 students (29.2%, SD = 4.7) watched videos to completion. For mini‐lecture videos containing the bulk of course material, these numbers declined to 8.3 student views (31.9%, SD = 3.5) at the beginning and 6.4 student views (24.6%, SD = 2.9) at the end of each video (Figure 1). These numbers reflect two important points: first, that the majority of students never began viewing the >30 mini‐lecture videos whose primary purpose was to reinforce material from the textbook, which likely led to considerable gaps in student understanding; and second, that some students who began watching these videos did not watch them to completion.

The content of on‐demand videos influenced whether students chose to view each video. Significant differences were found between the number of views at the beginning of each of three video types (i.e., mini‐lecture, example problem, or answer key; *F*(2, 43) = 37.59, *p *< .01), with by far the greatest average number of views at the beginning of the video observed for the example problem videos (17.2 views, or 66.2%, SD = 3.3) (Figure 1). In fact, the average number of views at the beginning of example problem videos was more than twice as high as that of mini‐lecture videos and more than 10 times greater than that of answer key videos (Figure 1). This indicates that students were more likely to watch videos that were necessary for completing problem sets than they were to watch videos pertaining to textbook content or to the correct answers to graded problems. This suggests that during this emergency online teaching period, student interest in understanding course content may have dwindled and may have been overshadowed by their desire to receive a passing grade, even if their understanding was lacking.

Further, the content of on‐demand videos influenced whether or not students watched videos to completion. When data were paired by video type, significant differences were found between the average number of views at the beginning and end of video for both mini‐lecture videos (*F*(10, 20) = 10.67, *p *< .01), but not for answer key videos, which each had fewer than five views, or for example problem videos. Nevertheless, these results indicate that a number of students who began watching these videos did not watch the videos to completion. In fact, it was occasionally observed that students would skip to certain parts of each video, skimming the content and likely focusing on what they deemed to be most important or on the topics that they found to be most interesting. It is also noteworthy to recognize that the average number of views at the beginning and end of the example problem videos were not found to be significantly different (*F*(7, 3) = 5.98, *p* = .08), indicating that more students tended to remain engaged in watching throughout the length of these videos, which were often necessary to view in order to successfully complete problem sets. This further supports the claim that student motivation and engagement during this emergency online learning period may have been more strongly influenced by measurable metrics of success (i.e., good grades) rather than a robust understanding of course material.

**FIGURE 2:**
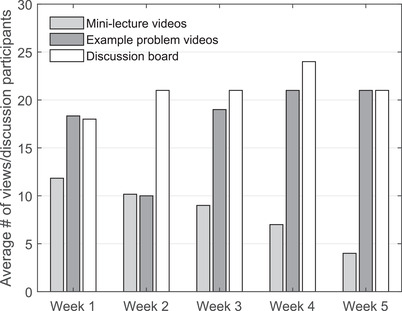
Average number of video views and total number of discussion board participants per week during the emergency online learning period. Statistically significant declines were observed in the average number of mini‐lecture videos each week, no statistically significant differences were observed in the average number of example problem video views each week, and no statistically significant differences were seen in the number of weekly discussion board posts when the second week was removed as an outlier

In addition to low average mini‐lecture video views, the number of mini‐lecture video views declined significantly each week during the online learning period, from an average of 11.8 views (45.4%, SD = 3.1) on Week 1 to an average of only four views (15.4%, SD = 1.2) on Week 5 (Figure 2). Statistically significant declines (*F*(2, 26) = 11.25, *p *< .01) in the number of student mini‐lecture video views were found between each consecutive week during this period. On the other hand, the average number of views of example problem videos were high and remained high throughout the course of the online learning period, with the notable exception of the second week, when no problem set was due. When the second week was removed as an outlier, no statistically significant differences in the number of example problem videos views were found (*F*(3, 2) = 8.67, *p *= .11) between the remaining weeks. These findings provide additional support for the idea that student participation and motivation during this period were primarily driven by a desire to pass the course rather by an interest in learning.

**FIGURE 3:**
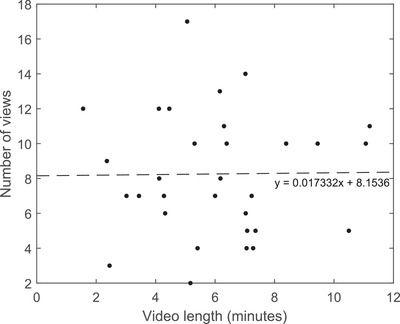
Average number of views of mini‐lecture videos vs. video length. The dashed line is the line of best fit and indicates that there is not a strong relationship between the average number of student video views and the length of the video

Unlike the results found by previous studies (e.g., McGowan & Hanna, 2015), who have observed consistent declines in student views as video length increases, the number of student views per mini‐lecture video did not tend to increase or decrease as video length increased (Figure 3). On‐demand mini‐lecture videos available to students ranged in length from 1 minute 39 seconds to 11 minutes 20 seconds (*n* = 31), and my results indicate that there was not a strong relationship between video length and the number of student views per video. These findings may be a result of the relatively short duration of the videos, which were purposefully made short to encourage student viewing. However, despite the fact that most videos were well within the 10‐minute limit suggested by McGowan and Hanna (2015), on average fewer than 40% of students watched the videos.

### 3.2 Student participation in discussion boards

Unlike the low student engagement observed for on‐demand mini‐lecture videos, student participation in weekly graded discussion boards was high, with an average of 21 students (80.8%, SD = 2.1) earning full participation points each week and with participation levels remaining steady throughout the online learning period (Figure 2). The high number of discussion participants each week mirrors the high number of example problem video views each week, again with the exception of the number of example problem video views in Week 2, when no problem set was due. This strengthens the hypothesis that students were more strongly motivated to actively engage in online coursework when that engagement was directly related to their grade in the course, an idea that has been corroborated by past studies (e.g., Aloni & Harrington, 2018). Further, the average number of participants in weekly discussion boards surpassed the average number of views of example problem videos in most weeks, suggesting that students are even more willing to participate in graded (vs. non‐graded, such as on‐demand mini‐lecture videos) online work that requires less time and is less stringently graded than problem sets.

### 3.3 Relationship between online participation and final exam grades

**FIGURE 4:**
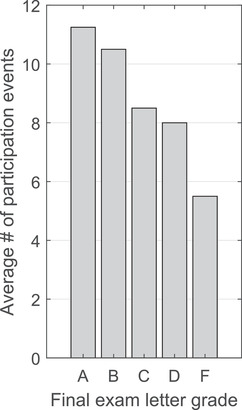
Average number of participation events per student during the 5‐week online learning period grouped by students’ final exam letter grade. Statistically significant differences were found for each letter grade.

Finally, I found that student final exam grades increased significantly as student online participation increased. In this case, a student online participation event was defined as either posting in a discussion board or submitting an online assignment. Statistically significant differences (*F*(4, 21) = 3.31, *p *< .05) were found between the average number of student online participation events by final exam letter grade (Figure 4), with students receiving As on the final exam averaging 11.5 participation events over the 5‐week online learning period, as compared to the 5.5 participation events on average for students who earned Fs. It is important to note that although final exams in this course rely upon students’ understanding of prior course material, they are not cumulative and thus were comprised entirely of material taught during the online learning period. These findings provide evidence that there is a strong relationship between student online participation in discussion boards and problem set completion and students’ final exam grades and demonstrate that students who were more engaged in online coursework were more likely to earn a higher grade on their final exam than were students who were less engaged.

### 3.4 Potential barriers to student online participation

It is important to keep in mind that in addition to the abrupt and unexpected transition to the online learning environment, student participation in online coursework was likely influenced by a host of outside factors caused by the COVID‐19 pandemic, including unreliable internet connections, economic hardships, changing work schedules, uncertain housing arrangements, the need to provide care for children no longer able to attend school or daycare, and increased anxiety, among many others. It is probable that every student faced one or more of these challenges, and that their engagement with course materials may have been negatively affected. These factors are, and will continue to be, important for instructors to consider when developing future online courses, especially in response to emergency situations.

### 3.5 Lessons learned and suggestions for future emergency online teaching

My findings provide important and relevant insights into student behavior and participation during compulsory online learning, and consideration of these trends should be taken when designing future online courses in response to COVID‐19 and other emergencies. My results suggest that, if possible, instructors should avoid an entirely asynchronous teaching model, which has been shown here to lead to low levels of student participation. Instead, instructors should attempt to maintain as much active contact as possible with students, whether that be by the use of online office hours, weekly online video discussion groups, or other online educational technology. It is likely that even one weekly synchronous online class meeting would lead to increased student engagement, as this meeting would provide a much greater opportunity for students to interact with one another and with the instructor than does a purely asynchronous teaching model.

Additionally, it is advisable that all university instructors be trained on the most fundamental aspects of their university's online learning environment prior to the beginning of instruction, regardless of the course delivery method the instructor intends to use. It is also advisable that instructors have some rudimentary plans in place for moving their courses online, should the necessity arise. This proactive stance would better prepare instructors to cope with the fast turn‐around times associated with emergency online learning in the future, which in the case of COVID‐19 were as short as 1 week.

## 4 CONCLUSION

The findings shown here indicate that compulsory online learning during the spring 2020 COVID‐19 emergency in the United States led to low levels of student participation in accessing ungraded, asynchronous course content, and that on average, fewer than 40% of students watched on‐demand course videos. However, student engagement in graded course content was shown to be consistently high throughout the online learning period. These results suggest that students were likely more motivated to participate online to earn points rather than to fully understand course material during the COVID‐19 emergency online learning period. My results also show that the overall number of mini‐lecture video views was low and not correlated with the length of the videos. Finally, and perhaps most importantly, my findings demonstrate that student online participation was positively correlated with increased student success as measured by final exam grades.

The continual, significant declines in student views of mini‐lecture videos throughout the online learning period shown here exemplify the thoughts of one student, who stated in their end of instruction course evaluation that “I really enjoyed this class until we went online, then I struggled to understand and no longer wanted to participate.” This sentiment is likely not unique to a single student or university, and it is the duty of universities and instructors to continue to improve online coursework to the best of their abilities so that students are actively engaged and remain engaged throughout the duration of the course, both during the ongoing COVID‐19 crisis and in potential future emergencies that may necessitate online learning.

## AUTHOR CONTRIBUTIONS

Briana Wyatt: Conceptualization; Data curation; Formal analysis; Investigation; Methodology; Visualization; Writing–original draft; Writing–review & editing.

## CONFLICT OF INTEREST

The authors have no conflicts of interest that would interfere with the fully transparent and objective presentation of this work.

## References

[nse220036-bib-0001] Aliyeva, D. M. (2020). The purpose of post‐reading activities in teaching. Science and Education, 7, 383–387. Retrieved from http://www.openscience.uz/index.php/sciedu/article/view/537

[nse220036-bib-0002] Aloni, M. , & Harrington, C. (2018). Research based practices for improving the effectiveness of asynchronous online discussion boards. Scholarship of Teaching and Learning in Psychology, 4(4), 271–289. 10.1037/stl0000121

[nse220036-bib-0003] Beaunoyer, E. , Dupéré, S. , & Guitton, M. J. (2020). COVID‐19 and digital inequalities: Reciprocal impacts and mitigation strategies. Computers in Human Behavior, 111, 106424 10.1016/j.chb.2020.106424 32398890 PMC7213963

[nse220036-bib-0004] Bristow, D. , Shepherd, C. D. , Humphreys, M. , & Ziebel, M. (2011). To be or not to be: That isn't the question! An empirical look at online versus traditional brick‐and‐mortar courses at the university level. Marketing Education Review, 21(3), 241–250. 10.2753/MER1052-8008210304

[nse220036-bib-0005] Crawford, J. , Butler‐Henderson, K. , Rudolph, J. , Malkawi, B. , Glowatz, M. , Burton, R. , & Lam, S. (2020). COVID‐19: 20 countries’ higher education intra‐period digital pedagogy responses. Journal of Applied Learning and Teaching, 3(1). 10.37074/jalt.2020.3.1.7

[nse220036-bib-0006] Jungmann, S. M. , & Witthöft, M. (2020). Health anxiety, cyberchondria, and coping in the current COVID‐19 pandemic: Which factors are related to coronavirus anxiety? Journal of Anxiety Disorders, 73, 102239 10.1016/j.janxdis.2020.102239 32502806 PMC7239023

[nse220036-bib-0007] Lyke, J. , & Frank, M. (2012). Comparison of student learning outcomes in online and traditional classroom environment in a psychology course. Journal of Instructional Psychology, 39(3–4), 245–250.

[nse220036-bib-0008] Mann, F. D. , Krueger, R. F. , & Vohs, K. D. (2020). Personal economic anxiety in response to COVID‐19. Personality and Individual Differences, 167, 110233 10.1016/j.paid.2020.110233 32834283 PMC7330578

[nse220036-bib-0009] Mcgowan, A. , & Hanna, P. (2015). How video lecture capture affects student engagement in a higher education computer programming course: A study of attendance, video viewing behaviours and student attitude. eChallenges e‐2015 Conference, Vilnius, 2015 (pp. 1–8). 10.1109/eCHALLENGES.2015.7440966

[nse220036-bib-0010] Ochsner, T. E. , & Robinson, J. S. (2017). The impact of a social interaction technique on students’ confidence and competence to apply stem principles in a college classroom. NACTA Journal, 61(1), 14.

[nse220036-bib-0011] Strong, B. , Davis, M. , & Hawks, V. (2010). Self‐grading in large general education classes: A case study. College Teaching, 52(2), 52–57. 10.3200/CTCH.52.2.52-57

